# Adherence to the recommended timing of focused antenatal care in the Accra Metropolitan Area, Ghana

**DOI:** 10.11604/pamj.2019.33.123.15535

**Published:** 2019-06-18

**Authors:** Kwaku Asah-Opoku, Donne Kofi Ameme, Alfred Yawson, Chris Bambey Guure, David Ernest Mireku Aduama, Kareem Mumuni, Ali Samba, Ernest Tei Maya

**Affiliations:** 1 Department of Obstetrics and Gynecology, University of Ghana School of Medicine and Dentistry (UGSMD), Korle-Bu, Accra, Ghana; 2 Ghana Field Epidemiology and Laboratory Training Programme, University of Ghana, School of Public Health, Legon, Accra, Ghana; 3 Department of Biostatistics, School of Public Health, University of Ghana, Legon, Accra, Ghana; 4 Greater Accra Regional Hospital, Greater Accra Region Accra; 5 Department of Population, Family and Reproductive Health, University of Ghana, School of Public Health, Legon, Accra, Ghana

**Keywords:** Adherence, focused antenatal care, determinants, timing

## Abstract

**Introduction:**

The proportion of antenatal attendants in Ghana who had at least four antenatal visits increased from 78% in 2008 to 87% in 2014. However, it is not known whether these visits followed the recommended timing of focused antenatal clinic attendance in Ghana. We sought to assess the adherence to the clinic schedule and its determinants in the Accra Metropolis.

**Methods:**

A cross-sectional study was conducted. Face-to-face interviews were conducted with postpartum women. Multiple logistic regression was used in the analysis of determinants of adherence to the recommended timing of clinic attendance. A p-value of <0.05 was considered statistically significant.

**Results:**

Among 446 focused antenatal care clinic attendants, 378 (84.8%) had four or more visits. Among these, 101 (26.7%) adhered to the recommended clinic schedule. Women who adhered were more likely to have had education up to Junior High School [AOR=3.31, 95%CI (1.03-10.61)] or Senior High School [AOR=4.47, 95%CI (1.14-17.51)], or have history of abortion [(AOR=3.36, 95%CI (1.69-7.96)]. For every week increase in gestational age at booking at the antenatal clinic, respondents were 34% less likely to complete all four antenatal visits at the recommended times. [(AOR=0.66, 95% (0.60-0.73)].

**Conclusion:**

Majority of women receiving focused antenatal care in the Accra Metropolis have four or more visits but only about a quarter of them adhered to the recommended clinic schedule. Having high school education, history of abortion and early initiation of antenatal care were predictors of adherence to clinic schedule. Women should be educated on early initiation of antenatal care to enhance adherence.

## Introduction

Antenatal care, an essential component of safe motherhood programme, entails systematic medical supervision of the pregnant woman until she goes into labour or until she is delivered of her baby via elective caesarean section. It seeks to ensure that every wanted pregnancy results in safe delivery of a healthy baby and a good outcome for the mother as well [1]. Antenatal care helps in the identification of conditions that are associated with poor maternal and perinatal outcomes thereby helping to provide preventive and curative health services for the pregnant woman and the fetus [2]. Antenatal care is critical to delivering the appropriate medical care according to the patients risk status and also serves as an avenue to educate expectant mothers on nutrition, personal hygiene and birth preparedness as well as provide them with physical and psychosocial support. All these benefits dovetail into an opportunity to prevent or minimize complications during pregnancy, delivery and the puerperium and also prepares expectant mothers to take care of their children physically, psychologically and socially [3]. The main types of antenatal care are the traditional and focused antenatal care. The World Health Organization (WHO) has recently introduced the Positive Pregnancy Experience Module of antenatal care [4]. The traditional antenatal model consists of monthly visits for the first six months, a visit every two to three weeks for the next two months, and weekly thereafter until delivery [5]. The Ghanaian version had women paying monthly visits up to 28 weeks, fortnightly visits from 28 to 36 weeks followed by weekly visits until delivery [6]. The WHO introduced Focused Antenatal Care (FANC) in 2002 to replace the traditional one which involved many visits and was not evidence based. For FANC, pregnant women without any medical or other significant health-related risks, or pregnancy related complications, four antenatal visits at specific gestational ages with targeted interventions are enough to meet their antenatal needs [7]. Ghana adopted FANC in 2002. The first visit should occur before 16 weeks. The second visit is between 20 to 24 weeks. The third visit is between 28 to 32 weeks and the fourth visit at 36 to 40 weeks [6]. In Ghana, the government has exempted fees for four visits for women attending antenatal clinics and also for delivery care. This is to remove intra-facility cost of antenatal care as an impediment to antenatal care [8]. A minimum of four antenatal visits is used as a proxy for adequate antenatal visits in Ghana. The proportion of pregnant women in Ghana who had at least four antenatal visits increased from about four in five in 2008 [9] to about nine in ten in 2014 [10]. What is not known however is how many of these women adhered to the recommended schedule for all four visits and the factors associated with this adherence. We sought to determine the adherence to the schedule for FANC and its associated factors among antenatal clinic attendees in the Accra Metropolitan Area.

## Methods

**Figure 1:**
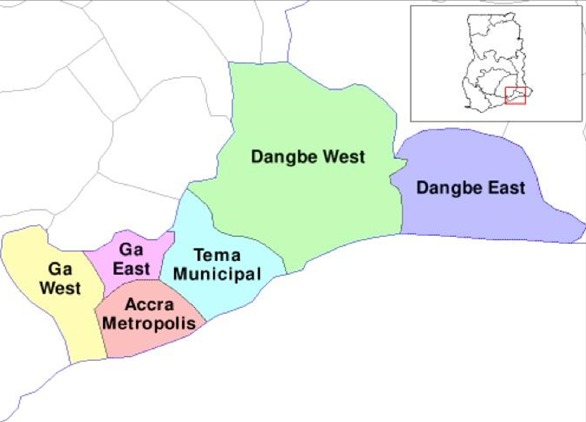
Map of the Greater Accra Region showing the Accra Metropolitan Area

A hospital based quantitative cross-sectional study was conducted in June 2017. Postpartum mothers from selected hospitals were interviewed on their clinic attendance for FANC. Data were also abstracted from health facility records to determine factors associated with adherence to the schedule for FANC. The study was conducted in the Accra Metropolitan Area (AMA), which is one of the 216 Metropolitan, Municipal and District Assemblies (MMDAs) in Ghana and among the sixteen MMDAs in the Greater Accra Region (Figure 1). The metropolis, is subdivided into ten sub metros. The population of the metropolis is 1,665,086 [11]. There are four public hospitals in the metropolis that provide maternity services. These are the Korle-Bu Teaching Hospital (KBTH), Achimota Hospital, Greater Accra Regional Hospital and the Maamobi General Hospital. All the facilities except KBTH were used for the study. The Korle-Bu Teaching Hospital was excluded because it does not conduct FANC as most of its clients are referrals with medical or obstetric risks. The annual deliveries for the Greater Accra Regional Hospital, Achimota Hospital and the Maamobi General Hospital in the year 2015, were 8432, 3000 and 1926 respectively. This gives a ratio of 6:2:1 annual deliveries. The study participants were first day postpartum mothers, who had had focused antenatal care at the three study sites. Postpartum mothers were recruited since they had completed their antenatal care by virtue of the fact that they have delivered. Postpartum day one was also chosen because the women were in the health facilities and therefore access to their antenatal booklets was easy. More importantly, it enabled the capture of women with poor outcome (e.g. still births) who, very likely, would not have come for postnatal services. Postpartum women whose maternal health records and delivery summaries could not be traced and those who were too ill to be interviewed were excluded. The minimum sample size of postpartum women required was calculated using the formula for cross-sectional study for an infinite population.

$$N=\frac{\left(z_{1-\alpha /2})2\;P(1-P\right)}{d^{2}}$$

Where, N = required sample size Z_1-α/2_ = confidence level of 95% (standard value of 1.96), p = percentage of postpartum women who attended all four antenatal visits at the recommended times was assumed to be = 50% = 0.5 (50% was used as it will produce the highest minimum sample size; since we are not aware of any study that captured the proportion of women with correct timing of all four antenatal visits), d = allowable margin of error = 5%. The minimum sample size calculated was 385 and adjusted upward by 20% for incomplete data and inconsistencies to 462. Data were collected over 28 days, hence, 17 study participants were randomly selected from the three hospitals per day. Using the ratio of their annual deliveries, 11:4:2 respondents were recruited daily from the Greater Accra Regional, Achimota and the Maamobi General hospitals respectively for interview. Study participants were recruited from the postnatal wards a day after delivery. Each day, the folders of all mothers who delivered the previous day and had FANC (identified through their antenatal records) were serially numbered. The serial numbers were then written on pieces of papers and put in an opaque envelope. The required daily numbers as indicated above, were picked blindly with replacement. If a number that had been picked already was picked up again it was replaced until a different number was picked. Face-to-face interviews were conducted by trained research assistants who also extracted data on antenatal care. Data were entered into Microsoft Office Excel 2007, imported and analysed with Stata Version 15 (Stata Corporation, College Station, TX, USA. The data entry sheet was designed with appropriate variable definition in place and consistency checks to minimize error during the data entry. The data were doubly entered by two trained research assistants. This helped in detecting any discrepancy by running frequency checks on both sets of data. The data were then cross checked and the necessary corrections were made for accuracy of the final entered data. Mean and standard deviation were calculated for continuous variables that were approximately normally distributed whilst median and range were calculated for continuous variables that were not normally distributed. Student's t-test was used to compare the difference between two means. Categorical variables were summarized as frequencies and proportions. Pearson's chi square test statistic was performed on categorical data to test association between socio-demographic factors, obstetrics and gynaecological history and adherence to timing of focused antenatal care. Multiple logistic regression was used in the analysis of determinants of adherence to the timing of focused antenatal care. Variables that were significant at the bivariate level were included in the logistic regression model. Odds ratio (OR) and 95% confidence intervals (CI) were used to test the strength of association. In all analysis, a p-value of <0.05 determined statistical significance.

The primary outcome of interest was adherence to the recommended timing of all four FANC visits by Ghanaian standards [6]. Socio-demographic characteristics and past obstetrics and gynaecological history were the independent variables of interest. Study participants were described as adherent to the FANC timing if they had at least four antenatal visits and had visits falling within all the four recommended time periods for FANC visits. Clients who had less than four visits, or had at least four visits but missed one or more visits at the recommended timing were classified as non-adherent. Clients who had less than 4 visits were excluded from the analysis of measures of association because the focus of the study was to determine whether the timing of antenatal visits by women who have four or more antenatal visits and would ordinarily be said to have had adequate antenatal care was appropriate. To ensure data quality and assure validity and reliability of the information obtained, three research assistants were trained over a 2 day period before pretesting of the questionnaire was carried out. Daily data validation meetings were also held. The questionnaire was pre-tested at the La General Hospital located in the La Dadekotopon Municipality, which adjoins the Accra Metropolitan Area. The La General Hospital serves a population with similar socio-demographic characteristics as the study hospitals. Ethical clearance to carry out the study was obtained from the Ghana Health Service Ethical Review Committee prior to commencement of the study. Administrative approval was obtained from the Greater Accra Regional Health Directorate and heads of the various hospitals. A written informed consent was obtained from each respondent aged 18 years and above. For respondents below 18 years, written informed consent was obtained from their parents/guardians and assent from those respondents.

## Results

Out of the estimated 462 study participants, 446 (96.5%) had been recruited at the end of data collection. They were all included in the data analysis. The mean age of all the respondents was 29.0 ± 5.8 years with majority 104 (23.3%) being 25 years and above. Most, 190 (42.6%) had an educational level up to Junior High School. Two hundred and sixty eight (60.1%) of the respondents were married. Majority, 376 (84.3%) of the respondents were employed and most, 337 (75.6%) were Christians (Table 1). Out of the 446, 68 (15.2%) had less than 4 antenatal visits, 277 (62.1%) had 4 or more visits but did not adhere to the timing of focused antenatal care visits while 101 (22.6%) had 4 or more visits and adhered to the recommended FANC timing. Out of the total of 378 (84.8%) respondents who had four or more antenatal visits, 101 (26.7%) adhered to all the four recommended timing of FANC. Out of the 446 respondents, 168 (38%) had been delivered once while 228 (51.1 %) had two or three deliveries. The median parity was 2 with a minimum of 1 and a maximum of 8. Majority, 435 (97.5%) of the respondents had no history of stillbirth. Seventy two percent have a history of abortion. Forty-one (26%) of the respondents had their previous pregnancies planned whiles 229 (51.3%) had planned their current delivery (Table 2). One hundred and fifty four (40.7%) of the 378 who had four or more antenatal visits had the first visit at the recommended time, 279 (73.8%) had the second visit at the recommended time, 338 (89.4%) had the third visit at the recommended time whiles 323 (85.4%) had the fourth antenatal visit at the recommended time. Out of the 378 (84.8%) of study participants who had four or more visits, 369 (97.6%) had at least one visit at the recommended time, 352 (93.1%) had at least two visits at the recommended times, 372 (72.0%) had at least 3 visits at the recommended times and 101 (26.7%) attended at least four of those visits at the recommended focused antenatal attendance times (Table 3). Among women who had four or more antenatal visits, there was no significant difference between the mean age of women who adhered to the timing (29.2 ± 6.1) and those who did not (29.5 ± 5.2) (p=0.711) likewise their other socio-demographic characteristics (Table 4). Among respondents who had 4 or more visits, a history of previous abortion was significantly associated with antenatal visits at the recommended times (χ^2^=14.724, p<0.001) (Table 4). Mean gestational age of booking of 11.2+/-2.9 weeks among women who had four or more focused antenatal care was significantly different from women who had less than four antenatal visits 19.1 +/- 6.1 weeks (t test=16.388, p<0.001). After adjusting for various confounders, the odds of correct timing of focused antenatal care in women who had completed Junior High School and Senior High School were 3.3 times [(AOR=3.31, 95% (1.03-10.61)] and 4.5 times [(AOR=4.47, 95% (1.14-17.51)] respectively higher than that of those without formal education for adherence to the recommended timing of focused antenatal care (Table 5). Compared to those without correct timing of focused antenatal care, women with correct timing had 3.7 times the odd of having previous abortion [(AOR=3.66, 95% (1.69-7.96)]. For every one week increase in gestational age at booking respondents were 34% less likely to complete all four antenatal visits at the recommended times [(AOR=0.66, 95% (0.60-0.73)] (Table 6).

**Table 1:** Socio-demographic characteristics of post -partum women who received focused antenatal care in the Accra Metropolitan Area in 2017

Variable	Frequency (N=446)	Percentages (%)
Age group		
<25	104	23.3
25+	342	76.7
**Educational level**		
No formal education	51	11.4
Primary	53	11.9
JHS	190	42.6
SHS	96	21.5
Tertiary	56	12.6
**Respondents occupation**		
Unemployed	70	15.7
Employed	376	84.3
Partner’s occupation		
Unemployed	29	6.5
Employed	417	93.5
**Marital status**		
Single	69	15.5
Cohabiting	109	24.4
Married	268	60.1
**Religion**		
Muslim	109	24.4
Christian	337	75.6
**Ethnicity**		
Akan	164	36.8
Ewe	72	16.1
Ga/Dangbe	89	20.0
Others^a^	121	27.1

a Other Ethnic groups included Guan, Hausa, Grusi, Dagomba, Frafra, Mossi, Fulani

**Table 2:** Obstetric and gynecological characteristics of post -partum women who received focused antenatal care in the Accra Metropolitan Area in 2017

Variable	Frequency ( N=446)	Percentage (%)
**Parity**		
1	168	37.7
2-3	228	51.1
4+	50	11.2
**History of stillbirth**		
No	435	97.5
Yes	11	2.5
**History of abortion**		
No	321	72
Yes	125	28
**Penultimate pregnancy**		
Unplanned	183	41
Planned	117	26
Not applicable	146	32
**Outcome of penultimate pregnancy**		
Live birth	273	61.2
Still birth/abortion	18	4.0
No previous pregnancy	155	34.8
**Satisfaction with care during ANC for penultimate pregnancy**		
Very satisfied	269	60.3
Moderately satisfied/not satisfied	33	7.4
No previous antenatal history	144	32.3
**Satisfaction with care during delivery at the penultimate pregnancy**		
Very satisfied	268	60.1
Moderately satisfied/not satisfied	25	5.6
No previous antenatal history	153	34.3
**Adequate help in taking care of baby at home following the penultimate pregnancy**		
Yes	274	61.4
No	18	4.0
Not applicable	154	34.5
**Place of delivery in penultimate pregnancy**		
Health facility	283	63.5
Non health facility	28	6.3
Not applicable	135	30.3
**Current pregnancy**		
Planned	229	51.3
Unplanned	217	48.7

**Table 3:** Socio-demographic characteristics of women who attended 4 or more focused antenatal visits in the Accra Metropolitan Area by adherence to timing of visits

Variable	No N=277	Yes N=101	Total N=378	X^2^	P-value
**Age group**				0.374	0.541
<25 years	63 (75.9)	20 (24.1)	83 (100.0)		
≥25 years	214 (72.5)	81 (27.5)	295 (100.0)		
**Educational level**				2.113	0.715
No formal education	37 (80.4)	9 (19.6)	46 (100)		
Primary	29 (70.7)	12 (29.3)	41 (100)		
JHS	114 (72.6)	43 (27.4)	157 (100)		
SHS	65 (74.7)	22 (25.3)	87 (100)		
Tertiary	32 (68.1)	15 (31.9)	56 (100)		
**Respondents Occupation**				0.099	0.870
Unemployed	42 (75.0)	14 (250)	56 (100)		
Employed	235 (73.0)	87 (27.0)	322 (100)		
**Partner’s occupation**				0.190	0.803
Unemployed	17 (77.3)	5 (22.7)	22 (100)		
Employed	260 (73.0)	96 (27.0)	378 (100)		
**Marital status**				1.088	0.591
Single	43 (78.2)	12 (21.8)	55 (100)		
Cohabiting	69 (70.4)	29 (29.6)	98 (100)		
Married	165 (73.3)	60 (26.7)	225 (100)		
**Religion**				0.148	0.787
Muslim	66 (71.7)	26 (28.3)	92 (100)		
Christian	211 (73.8)	75 (26.2)	286 (100)		
**Ethnicity**				0.910	0.833
Akan	108 (74.0)	38 (26.0)	164 (100)		
Ewe	41 (68.3)	19 (31.7)	72 (100)		
Ga/Adangbe	57 (74.0)	20 (26.0)	89 (100)		
Others	71 (74.7)	24 (25.3)	121 (100)		

**Table 4:** Past obstetric and gynecological history of post-partum women who attended four or more focused antenatal care in the Accra Metropolitan Area by recommended timing of visits

Variable	Four or more visits		Total N=378	X^2^	P-value
	No N=277	Yes N=101			
**Parity**				2.421	0.303
1	111 (77.6)	32 (22.4)	143 (100)		
2-3	133 (70)	57 (30.0)	190 (100)		
4+	33 (73.3)	12 (26.7)	45 (100)		
**History of stillbirth**				2.601	0.197^b^
No	270 (72.8)	101 (27.2)	371 (100)		
Yes	7 (100.0)	0 (0.0)	7 (100)		
**History of abortion**				14.724	<0.001
No	210 (78.9)	56 (21.1)	266 (100)		
Yes	67 (59.8)	45 (40.2)	112 (100)		
**Penultimate pregnancy**				3.219	0.208
Unplanned	107 (68.6)	49 (31.4)	156 (100)		
Planned	82 (78.1)	23 (21.9)	105 (100)		
Not applicable	88 (75.2)	29 (24.8)	117 (100)		
Outcome of pregnancy*				0.331	0.871
Live birth	170 (72.3)	65 (27.7)	235 (100)		
Still birth/abortion	11 (73.3)	4 (26.7)	15 (100)		
No previous pregnancy	96 (75.0)	32 (25.0)	128 (100)		
Satisfaction with ANC*				1.356	0.517
Very satisfied	171 (73.7)	61 (26.3)	232 (100)		
Moderately satisfied/not satisfied	18 (64.3)	10 (35.7)	28 (100)		
No previous antenatal history	88 (74.6)	30 (25.4)	118 (100)		
Satisfaction with care during delivery*				0.341	0.852
Very satisfied	166 (72.2)	64 (27.8)	230 (100)		
Moderately satisfied/not satisfied	16 (76.2)	5 (23.8)	21 (100)		
No previous antenatal history	95 (74.8)	32 (25.2)	127 (100)		
Adequate help in taking care of baby following delivery*				1.062	0.582
Yes	173 (73.3)	63 (26.7)	236 (100)		
No	10 (62.5)	6 (37.5)	16 (100)		
Not applicable	94 (74.6)	32 (25.4)	126 (100)		
Place of delivery*				0.022	1.000
Health facility	179 (73.4)	65 (26.6)	244 (100)		
Non health facility	18 (72.0)	7 (28.0)	25 (100)		
Not applicable	80 (73.4)	29 (26.6)	109 (100)		
**Current pregnancy**				0.000	1000
Planned	140 (73.3)	51 (26.7)	191 (100)		
Unplanned	137 (73.3)	50 (26.7)	187 (100)		

b Fishers exact test

* Refers to penultimate pregnancy

**Table 5:** Socio-demographic factors influencing correct timing of four or more focused antenatal visits among postpartum women in the Accra Metropolitan Area

Variable	Crude OR (95% C.I)	P-value	Adjusted OR (95% C.I)	P-value
**Age group**				
<25 years	1		1	
≥25 years	1.19 (0.68-2.10)		1.24 (0.48-3.22)	0.658
**Educational level**				
No formal education	1		1	
Primary	1.70 (0.63-4.59)	0.294	3.57 (0.91-14.08)	0.069
JHS	1.55 (0.69-3.48)	0.288	3.31 (1.03-10.61)	0.044
SHS	1.39 (0.58-3.34)	0.459	4.47 (1.14-17.51)	0.031
Tertiary	1.93 (0.74-4.99)	0.177	3.06 (0.71-13.14)	0.132
**Respondents Occupation**				
Unemployed	1		1	
Employed	1.11 (0.57-2.13)	0.753	1.95 (0.67-5.72)	0.221
**Partner’s occupation**				
Unemployed	1		1	
Employed	1.25 (0.45-3.50)	0.663	1.65 (0.35-7.83)	0.529
**Marital status**				
Single	1		1	
Cohabiting	1.50 (0.70-3.26)	0.299	2.16 (0.67-7.00)	0.200
Married	1.30 (0.64-2.64)	0.462	1.52 (0.50-4.63)	0.459
**Religion**				
Muslim	1		1	
Christian	0.90 (0.53-1.53)	0.701	0.38 (0.13-1.08)	0.069
**Ethnicity**				
Akan	1		1	0.283
Ewe	1.31 (0.68-2.54)	0.412	2.15 (0.80-5.81)	0.130
Ga/Adangbe	0.10 (0.53-1.87)	0.993	0.67 (0.25-1.83)	0.436
Others	0.96 (0.53-1.73)	0.894	0.99 (0.33-3.00)	0.987

**Table 6:** Obstetric factors influencing correct timing of four or more focused antenatal visits among postpartum women in the Accra Metropolitan Area

Variable	Crude OR (95% C.I)	P-value	Adjusted OR (95% C.I)	P-value
**Parity**				
1	1		1	0.209
2-3	1.49 (0.90-2.45)	0.121	2.13 (0.69-6.62)	0.190
4+	1.26 (0.59-2.72)	0.554	4.11 (0.85-19.82)	0.078
**History of stillbirth**				
No	1		1	
Yes	0 (0-0)	0.999	0.00 (0-0)	0.999
**History of abortion**				
No	1		1	
Yes	2.52 (1.56-4.07)	<0.001	3.66 (1.69-7.96)	0.001
**Penultimate pregnancy**				
Unplanned	1		1	0.637
Planned	0.61 (0.35-1.09)	0.093	0.66 (0.27-1.60)	0.358
No previous pregnancy	0.72 (0.42-1.23)	0.231	0.66 (0.06-7.54)	0.738
Outcome of pregnancy*				
Live birth	1		1	0.591
Still birth/abortion	0.95 (2.9-3.09)	0.934	0.28 (0.02-3.77)	0.337
No previous pregnancy	0.87 (0.53-1.43)	0.584	1.06 (0.21-5.28)	0.943
Satisfaction with ANC*				
Very satisfied	1		1	0.428
Moderately satisfied/not satisfied	1.56 (0.68-3.56)	0.239	2.25 (0.35-14.42)	0.392
No previous antenatal history	0.96 (0.58-1.59)	0.861	6.92(0.29-162.68)	0.230
Satisfaction with care during delivery*				
Very satisfied	1		1	0.777
Moderately satisfied/not satisfied	0.81 (0.29-2.30)		0.60 (0.06-5.63)	0.656
No previous antenatal history	0.87 (0.53-1.43)		0.37 (0.02-8.66)	0.533
Adequate help in taking care of baby following delivery*				
Yes	1		1	0.985
No	1.65 (0.58-4.72)	0.352	1.17 (0.17-8.05)	0.875
No previous pregnancy	0.94 (0.57-1.53)	0.789	1.14 (0.11-12.31)	0.917
Place of delivery*				
Health facility	1		1	0.983
Non health facility	1.07 (0.43-2.68)	0.884	1.12 (0.15-8.34)	0.913
No previous pregnancy	0.94 (0.60-1.66)	0.995	0.93 (0.09-9.44)	0.952
**Current pregnancy**				
Planned	1		1	
Unplanned	1.00 (0.64-1.58)	0.994	0.86 (0.40-1.83)	0.691
Gestational age at booking	0.70 (0.65-0.76)	<0.001	0.66 (0.60-0.73)	<0.001

* Refers to penultimate pregnancy

## Discussion

The proportion of women having focused antenatal care in the Accra Metropolitan area and having four or more visits was about 85%. Only about a quarter of the women who had at least these four visits adhered to all the four recommended times for clinic attendance. Four out of ten women who had four or more antenatal visits had their first visit before 16 weeks, about 75% had their second antenatal visit between 20 and 24 weeks, close to 90% of the respondents had their third visit between 28 and 32 weeks while 85% had their fourth visit between 36 and 40 weeks. Women who had correct timing of focused antenatal care were more likely to have had education up to Junior or Senior High School, or had a history of abortion. For every week increase in gestational age at booking at the antenatal clinic, respondents were 34% less likely to complete all four antenatal visits at the recommended times. This study found that close to 40% of the respondents had their first antenatal visit less than 16 weeks. This is less than that recorded in the GDHS 2014 which was 64%. The reason for this could be the fact that Accra being a metropolitan area may have more women engaged in formal employment who may not be able to easily take time off work to start antenatal care especially when they do not have problems in early pregnancy. This relatively late reporting for the first antenatal visit is detrimental and may lead to incomplete focused antenatal intervention schedule [12]. Late reporting for the booking antenatal visit has also been associated with women who do not receive adequate education on danger signs of pregnancy. This figure is however higher than the 25% of pregnant women in Uganda whose booking visit occurred before 16weeks [13]. This difference may be attributed to the differences in socio-demographic characteristics between Ghana and Uganda. The 40% respondents who had their booking visit before 16 weeks is better than the 64% of antenatal women who present for their booking visit in the third trimester that was recorded in Kenya [14]. The Kenyan study was however in a rural community and also a community survey was used where access to patients antenatal records may be a challenge.

The proportion of women attending focused antenatal care in the Accra Metropolitan area and having at least four visits was about 85%. This is similar to the 87% national figures for Ghana and [10] Vietnam [15] antenatal attendants but higher than the 48% recorded in Uganda [13]. In our study, only about a quarter of the women who had at least four antenatal visits and normally would have been classified as having adequate antenatal care attended clinics within all the four recommended time frames. This study has revealed that even though majority of women had at least four antenatal visits, just a few adhered to all the four recommended schedule of focused antenatal care. This may have an impact on the effectiveness of various interventions provided during various visits because the interventions in FANC are hinged to specific gestational ages to allow for effective remedies to be provided to women. This also calls for the reassessment of FANC as practiced currently in the Metropolis by the health authorities. There is also the need to take a second look at the criteria of at least four antenatal visits to connote adequate antenatal attendance. The average gestational age at booking from this study was about 17 weeks. It is better than that recorded in North Central Nigeria which was 19 weeks [16]. The reason for this difference however is not clear. It is also better than the gestational age at booking of 20 weeks recorded in rural south eastern Tanzania [17]. This difference may be attributable to the fact that the latter study was carried out in a rural district where the socio-demographic characteristics of the respondents are likely to be different from this study, which was undertaken in an urban area. This rural urban difference also agrees with work done in Pakistan where urban women were found to initiate antenatal care early compared to those in rural areas [12].

From this study, the most significant determinants of adherence to the recommended timing of all four focused antenatal visits were women with either junior or senior high school educational level, a previous history of abortion and early initiation of antenatal care. With reference to education as a determinant for adherence to timing of focused antenatal care among women with four or more antenatal visits, the most significant determinant was women whose educational background was the Senior High School Level (p=0.031). This finding agrees with work done in Pakistan, which showed that women with secondary education had an odds of 5 times higher than that of those without formal education for adherence to the timing of focused antenatal care [18]. The likely explanation for this trend is that women with tertiary education are likely to be engaged in formal jobs, which may not enable them to attend antenatal care at the prescribed periods. Women with secondary education are likely to be involved in informal jobs and therefore may be able to easily make time to attend focused antenatal care. Women with a previous history of abortion are likely to have all four recommended focused antenatal visit timing right because they may want to prevent any mishap from happening in the index pregnancy. We cannot tell whether the effect of abortions on correct timing of antenatal care was due predominantly to those that were spontaneous or induced as we did not ask that specific question. These findings agree with the studies in rural south eastern Tanzania [17] where previous poor pregnancy outcomes (e.g. miscarriage, stillbirth) were associated with early antenatal booking. Women who booked early were more likely not to have missed the recommended timing for the first FANC and therefore likely to have all four recommended timing of visits correct compared to those who booked late. This study also found that among women who had at least four antenatal visits, the odds of adhering to all four recommended times among women who were employed was twice that of the unemployed; however this was not statistically significant (p=0.221). In Ghana, women who are employed are more likely to adhere to antenatal care guidelines compared to the unemployed [18]. In Pakistan, women who are employed have been found to be more likely to adhere to timing of focused antenatal care [19]. This study did not record this finding as statistically significant probably because of the limited number of health facilities used. There may be the need to carry out this study on a wider scale that may include many more centres.

Again from this study, among women who had four or more visits, those who were moderately or less satisfied with the care given to them in their previous delivery had an odds twice higher than that of those who were satisfied for observing the recommended timing of focused antenatal care. This finding was however not significant. Other studies had showed that poor healthcare provider attitude in previous antenatal experience was associated with poor adherence to timing of focused antenatal care [17, 20]. There may be the need to examine where the previous antenatal visits during which clients were not satisfied were held and whether they had changed facilities for antenatal care to be able to understand this phenomenon. This was not looked at in this study. Amoakoh-Coleman *et al* (2016) observed that women who were less than 25 years were more likely to initiate antenatal care early [18]. This study found out that among postpartum women who had four or more focused antenatal visits, those aged 25 or more had approximately an odds of 25% more to adhere to FANC timing than that of those less than 25 years, but this was not a statistically significant finding. Regarding planning of pregnancy as a predictor of adherence to timing of focused antenatal care, previous studies had produced conflicting results. While in Brazil unplanned pregnancies were significantly associated with adherence to the timing of focused antenatal care [21], in Rwanda planned pregnancies rather were associated with adherence to the timing of first antenatal care [22]. Our study did not show any statistically significant association between planning of pregnancy and adherence to all four antenatal schedule. With regard to knowledge about recommended timing for the first antenatal attendance almost three out of four of the respondents knew about the fact that the first antenatal visit should be at gestational age less than 16 weeks. This is similar to results obtained in Uganda [23]. This knowledge however did not translate into practice as less than half of the respondents actually reported for their first antenatal visit before 16 weeks. The strength of our study is that, to the best of our knowledge, it is the first study in Ghana that has looked beyond just four antenatal visits and considered the accurate timing of clinic attendance. Our study had some limitations. First, we relied on clinical records that were entered by many people and therefore there is the possibility that some of the data may not be accurate. In addition, it is possible participants gave more favourable responses regarding questions about satisfaction with care during antenatal and previous deliveries in order not to offend their care givers.

## Conclusion

This study has shown that while over eight out of ten women receiving focused antenatal care in the Accra Metropolitan area have at least four visits, only about one out of five of those with at least four visits adhered to all the four recommended timing of attendance. Early initiation of antenatal care, an educational level up to Junior or Senior High School as well as a history of abortion were predictors for adherence to all four timings of focused antenatal care. There is the need to educate women on early initiation of antenatal care to enhance full adherence. Reassessment of the criteria for adequate antenatal care in Ghana must be considered.

### What is known about this topic

- Evidence-based focused antenatal care reduces number of antenatal visits and cost of care;
- Four or more antenatal visits is an indicator for adequacy of antenatal care.

### What this study adds

- Majority of pregnant women who practice focused antenatal care do not adhere to the recommended timing;
- Early initiation of antenatal attendance and previous abortion are determinants of adherence to the recommended timing of focused antennal care.

## Competing interests

The authors declare no competing interests.
